# Vertical and horizontal reading training in patients with hemianopia and its effect on reading eye movements

**DOI:** 10.1038/s41598-024-52618-y

**Published:** 2024-02-12

**Authors:** S. Kuester-Gruber, P. Kabisch, A. Cordey-Henke, P. Martus, H.-O. Karnath, S. Trauzettel-Klosinski

**Affiliations:** 1 Vision Rehabilitation Research Unit, Center for Ophthalmology, University of Tübingen, Tübingen, Germany; 2 Institute for Clinical Epidemiology and Applied Biostatistics, University of Tübingen, Tübingen, Germany; 3 Center of Neurology, Division of Neuropsychology, Hertie-Institute for Clinical Brain Research, University of Tübingen, Tübingen, Germany

**Keywords:** Translational research, Brain injuries, Stroke, Vision disorders, Brain injuries, Stroke

## Abstract

Vertical reading training (VRTr) increases reading speed (RS) significantly in patients with hemianopic field defects (HFD). We ask, how eye movements (EM) contribute to this improvement and whether EM-behavior is affected by the side of HFD. Twenty-one patients, randomly assigned to VRTr or horizontal RTr, trained reading single lines from a screen at home, for 4 weeks. In the clinic, we recorded EM while reading short sentences aloud from a screen before training (T1), directly (T2) and 4 weeks afterwards (T3). RS-screen was correlated with RS during reading printed paragraphs (RS-print) to assess the transfer to everyday life. RS-screen and RS-print correlated positively (horizontal: r > 0.8, vertical: r > 0.9) at all times. Vertical RS did not exceed horizontal RS. We found significant negative correlations of EM-variables and RS-print: in right-HFD with the number of forward saccades (T1: r =  − 0.79, T2: r =  − 0.94), in left-HFD with the steps during return sweeps (T1: r =  − 0.83, T2: r =  − 0.56). Training effects remained stable at T3. EM-improvement was specific for the RTr and the side of the HFD: in right-HFD fewer forward saccades after VRTr, in left-HFD fewer steps during return sweeps after HRTr. RTr on a screen transfers to reading printed text in real-life situations.

Trial registration: The study was retrospectively registered in the German Clinical Trials register: DRKS-ID: DRKS00018843, March 13th, 2020.

## Introduction

Hemianopic field defects (HFD) can cause severe reading disability. For left-to-right readers, right hemianopia is much more disabling, because they have to perform reading saccades into the scotoma. This was first described by Mauthner 1881^1^ and later confirmed by other authors^2–7^. A few eye movement studies have shown that patients with right HFD make many small saccades in reading direction and more backward saccades to get through the line^5–9^. Patients with left HFD have no problem reading through the text on one line, but need additional backward saccades during the return sweep, to find the beginning of the next line^6^.

**Figure 1:**
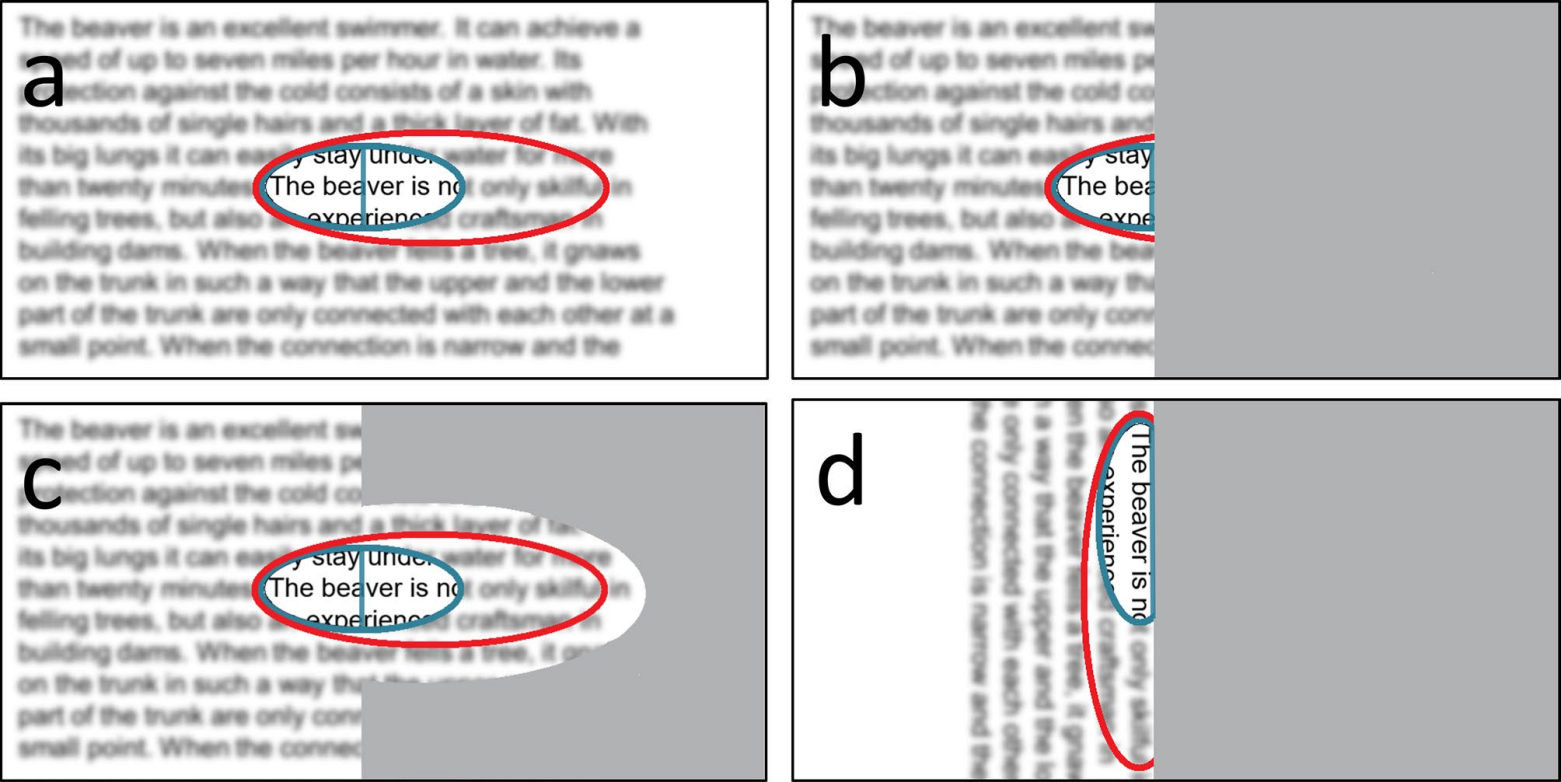
Macular sparing and reading. (**a**) The blue oval shows the visual span, or minimum reading visual field, where the letters are seen clearly (2° to the right and left of fixation)^27,28^. The red oval shows the perceptual span, which can be extended in reading direction up to 5°^16^. (**b**) With macular splitting half of the visual span is lost. (**c**) With macular sparing (> 5°) reading is not impaired. **d**) Can the seeing visual field at the margin of the scotoma be utilized by vertical reading?

The question whether a hemianopic dyslexia occurs depends on the presence and size of a macular sparing (Fig. 1c). The existence of a macular sparing had been discussed controversially over decades, for an overview see^10^. It was assumed to be a perimetric artefact due to fixational eye movements during perimetry, which cause a shift of the field defect towards the hemianopic side^11^. We showed in a study based on microperimetry using a Scanning Laser Ophthalmoscope with a 1° test-point grid that macular sparing exists^12^ and that it is associated with sparing of the occipital pole in lesions of the primary visual cortex.

This central seeing area on the hemianopic side is preserved by a double blood supply in patients with hemianopia from stroke (after occlusion of the posterior cerebral artery), if the middle cerebral artery supplies the occipital pole, where the macula is represented. The size of the macular sparing varies dependent on the individual anatomical conditions, but never exceeds 10°^10,13^. The prevalence of a macular sparing was reported in a retrospective study based on a cohort of 904 HFD patients with 7.3%^14^. This low percentage might be due to a high portion of patients with traumatic brain injury. On the other hand, one has to consider that perimetric artefacts can mimic sparing due to unstable fixation, and often the visual field is not examined with sufficient precision in clinical settings. The true prevalence is not known and depends on the variable individual anatomical conditions^10^.

We have previously shown that a macular sparing of > 5° is necessary for unimpaired reading in HFD^6,12,15^. This confirmed the results about the perceptual span that is necessary for fluent reading in healthy subjects^16^. McConkie and Rayner found the perceptual span during one fixation asymmetric to the right in left-to-right readers (larger in reading direction)^17^. We found in patients with left HFD that a perceptual span of > 5° is required also for the return sweep to the next line^6^. A later study confirmed our finding in simulated hemianopia regarding the word-length effect on reading onset time depending on the size of the “macular sparing”^18^.

Another feature that should be differentiated from macular sparing, is a “nasotemporal overlap”^19^ of retinal ganglion cells that leads to a strip of perception of 0.5–1° in the contralateral field along the vertical meridian, but not in the macular area.

The first training studies were about reading moving text^4,5,20^ and oculomotor training using single words and non-word units^8^, which was reported to improve reading. There are a few RC-studies on reading training in patients with HFD: Scrolled text improved RS in right HFD^21^ and search tasks in a line of text increased RS in left HFD more than in right HFD^22^.

Whereas previous treatment methods focused on oculomotor training, we used a new approach here**:** We investigated whether patients with HFD can utilize an area of the seeing visual field along the vertical border of the scotoma by turning the text by 90 or 270 deg and then reading in a vertical direction, which can overcome the problem of a limited perceptual span (Fig. 1). Figure 1 shows the conditions of the visual and perceptual span on the text and demonstrates the importance of the perceptual span for undisturbed reading. Studies in healthy subjects on reading in a vertical direction found reading to be much slower than in the horizontal direction, for languages that are usually read horizontally, e.g. English, German, Finnish^23–26^. This has been attributed to a smaller visual span^27,28^ in the vertical axis, to a slightly steeper decline of resolution along the vertical meridian^29^, and to increased crowding^28,30^. Other studies found that healthy subjects improved only after 10 weeks of training^26^. A study with healthy subjects, who trained vertical reading text presented as single words (Rapid Serial Visual Presentation, RSVP), showed on average no statistically different reading speed between vertical and horizontal reading speed after 4 days of training, but 5 of the 6 individuals were still faster reading horizontally^31^. The authors mention a potential of vertical RSVP-training for patients with visual field defects involving the central visual field.

A study on patients with age-related macular degeneration, who had their scotoma shifted in reading direction, showed an improvement of reading speed of text presented as RSVP and rotated by 90°, but this was slower than reading in a horizontal direction^32^.

We conducted a pilot study with healthy subjects and also found that reading in a vertical direction was slower than in the horizontal direction. Furthermore, the participants reported that they had to concentrate more on the unfamiliar word display than on text comprehension^33^.

Whereas healthy subjects will not have a perceptual advantage by turning the text, patients with HFD might benefit from reading without being limited by their scotoma. A few studies examined vertical reading in hemianopia and found slower reading speeds^34,35^. However, none of the previous studies on patients with HFD applied training of vertical reading.

Recently, we performed the first study of training to read vertically oriented text in patients with HFD^33,36^. The results showed that reading training in vertical text orientation improved reading speed (RS) and quality of life significantly, mainly in patients with right HFD. On the other hand, reading training in horizontal text orientation (from left to right) improved RS significantly, mainly in patients with left HFD. The improved reading speed during vertical reading did not reach the speeds achieved during horizontal reading.

In the previous study^36^, we focused on the effect of the training on reading speed.

In the current study, we analyze the reading eye movements (EM). Thus, the aim of the current study was to clarify the contribution of EM to the improvement of RS after training in both reading directions. Furthermore, we examined whether the training affects EM behavior specifically in patients with left or right HFD. First results have been reported before^37^ .

Furthermore, we were interested in detecting pre-existing or newly induced adaptations of EM behavior. For this reason we used a scanning laser ophthalmoscope (SLO) as a supplement to the EM analysis by an infrared eye tracker. The SLO visualized the EM directly on the retina during different tasks^12,38,39^. Adaptations mean unconscious compensative strategies by the patients – oculomotor and sensory—without training in order to overcome the problem of the reduced perceptual span.

Our previous studies had shown that some patients with hemianopia without macular sparing can use a slightly eccentric (1–1.5°) fixation locus despite normal visual acuity^12,38^. This adaptive behavior creates a small perceptive area along the vertical midline that can be used for reading. In a previous study we showed^12^ different fixation patterns dependent on the size of macular sparing: If the sparing was 5°, fixation was central and stable. The smaller the macular sparing (2–4°), the less stable the fixation. In macular splitting (0°) we found either central unstable fixation with frequent saccades towards the hemianopic side or eccentric fixation, both resulting in a shift of the field defect in perimetry towards the hemianopic side.

Another adaptive behavior is the use of hypermetric/predictive saccades to find the next line in left HFD and preview the line in right HFD^39–41^. For the early stages of HFD, Meienberg and colleagues^40^ described a staircase pattern of the EM caused by a series of saccadic movements towards a target on the blind side at a distance of ± 10° from the center. The authors call it “safe but slow”. Once the target position had been learned by several trials, the patients performed overshoot saccades to find the target, or predictive saccades, dependent on the target presentation.

This behavior was a short-time adaptation during one session. (One of 3 patients was followed up after 7.5 months. He had changed his performance and made one large saccade that overshot the target). In a later study, Meienberg^41^ confirmed in 12 patients with right and left HFD that these EM patterns for finding targets (staircase, overshoot, predictive) were independent of the time since onset of the HFD. We conducted an SLO-study^39^, where we examined dysmetric saccades to a target (± 5° apart from the central fixation cross) and found in principle the same behavior. We showed that dysmetric saccades to targets (5° apart) occurred more frequently in patients without macular sparing (< 4°) than in those with a sparing =  > 4°. We did not find a correlation with disease duration (0.2–29 years), which indicates insufficient spontaneous long-term adaptation. The studies mentioned above did not examine the saccadic adaptation behavior during reading. This is why examining these pre-existing spontaneous adaptations and their potential influences on the training effect (and vice versa) were of interest in the current study.

We hypothesize that the improvement of reading speed in either text orientation will be influenced by specific changes in EM patterns dependent on the side of the HFD. We also anticipate that training to read single lines of text on a screen will to some extent transfer to reading printed text in a real-life situation.

## Methods

### Study design

Patients were randomly assigned to either of two training groups: Group V (n = 11) to train reading in vertical text orientation, and group H (n = 10) to train reading in horizontal text orientation. The horizontal training was supposed to be a placebo training and served as a control.

Examinations were performed before training (T1), directly after end of training (T2) and after four weeks without training (follow-up, T3).

### Patients

We screened a large sample of 862 patients with visual field defects due to brain damage of different pathologies. Due to the strict inclusion and exclusion criteria (see below), only 36 of these 862 patients with HFD could be recruited.

Of these, 15 had to be dropped, for a variety of reasons: They either did not show up after training (n = 5), had insufficient training intensity (n = 3), aphasia (n = 2), suspected alexia (n = 2), developmental dyslexia (n = 1), AMD (n = 1), or for personal reasons (n = 1).

Finally, 21 patients with hemianopia (left, n = 11; right, n = 10, one of them with upper right quadrantanopia) participated in the study. The examinations were conducted between 07 March, 2016 and 20 August, 2018.

The detailed clinical data at baseline are shown in Table 1. There was no difference between the groups regarding age, disease duration, side of the HFD, size of macular sparing, reading speed of printed paragraphs, or cognitive ability.

**Table 1:** Demographic and clinical data at baseline.

	Group V	Group H	∆
n	11	10	
Age [years]	58.88 (12; 40.25–80.17)	65.95(8.3; 57.25–81.17)	*p* > 0.05
Cause			
Stroke	4	6	
Hemorrhage	1	2	
Tumor	3	2	
Trauma	2	0	
Duration of disease [years]	5.1 (7.75; 0.5–26.00)	5.9 (6.72; 1.00–19.00)	*p* > 0.05
HFD right	6	4	
HFD left	5	6	
Macular sparing [°]	1 (1.55; 0–5)	0.9 (1.2; 0–4)	*p* > 0.05
RS-print at baseline (IReST) [wpm]	95.57 (36.26; 31–144)	102 (32.27; 45–146)	*p* > 0.05
MoCA	24.55 (3.39; 20–30)	23.70 (3.34; 19–29)	*p* > 0.05

*RS-print* reading speed of printed paragraphs at first visit, *wpm* words per minute, *MoCA* Score in the Montreal Cognitive Assessment Test.

Means (standard deviation; min–max).

All patients had a macular sparing of ≤ 5 degrees, which was an inclusion criterion (see below). Macular splitting was found in 10 patients, a sparing of 1° in 7 patients, of 2° in 2 patients, of 4° and 5° in one patient each.

Inclusion criteria:

- HFD since ≥ 6 months to exclude interference with spontaneous recovery
- macular sparing of ≤ 5 degrees to include only patients with limited perceptual span
- normal or nearly normal cognitive ability (assessed by the Montreal cognitive assessment (MoCA), (MoCa scale =  > 18)^42^
- visual acuity of at least 0.6 (0.2 LogMAR)
- Reading speed < 150 words per minute
- German as native language
- Ability to consent

Exclusion criteria:

- Additional visual field defects
- Any eye disease, except mild cataract
- Spatial neglect (assessed by line bisection test)
- Lack of interest in reading
- Insufficient co-operation
- Aphasia, alexia
- Reading impairment of other origin

These strict inclusion and exclusion criteria were chosen to include only patients without macular sparing (≤ 5°), who have a reduced perceptual span, and to make the final group as homogeneous as possible, which also made recruitment very difficult.

### Patient examinations

#### Clinical examinations at baseline

Clinical neuro-ophthalmologic and orthoptic examinations were performed in all patients, including perimetry (30°), tests of best-corrected visual acuity (far/near), binocular status, motility, and eye morphology.

Microperimetry was performed to assess the size of macular sparing by semi-automated, custom-designed software for an SLO with a test grid of 1 degree, performed monocularly on both eyes.

#### Specific examinations

An overview of all procedures and measurements of variables is shown in Table 2.

**Table 2:** Overview on the procedures, measurements of variables and correlations at T1, T2, T3.

Reading speeds (analyzed according to reading direction, group and side)	RS-print-H: paragraphs
	RS-print-V: paragraphs
	RS-screen-H: sentences
	RS-screen-V: sentences
Correlations of RS	RS-print-H versus RS-screen-H
	RS-print-V versus RS-screen-V
EM variables (horizontal reading, analyzed according to side of HFD)	Side of HFD
	Number of forward saccades per word
	Number of backward saccades per word
	Number of additional steps during the return sweep
	Fixation duration
Correlations between EM variables during horizontal reading and RS-print- H	
SLO: adaptations	Fixation locus (central/eccentric)
	During fixation of a cross
	During reading
	Dysmetric saccades

*RS-print-H* Reading speed during horizontal reading of printed paragraphs, *RS-print-V* Reading speed during vertical reading of printed paragraphs, *RS-screen-H* Reading speed during horizontal reading of sentences on the screen, *RS-screen-V* Reading speed during vertical reading of sentences on the screen, *HFD* hemianopic field defect, *EM* eye movements.

#### Eye movements during reading short sentences on a screen

Eye movements (EM) were recorded by an infrared limbus tracker (JAZZ-novo, Ober Consulting, Poznan, Poland) with a sampling rate of 1000Hz and a spatial resolution of 0.1°. EM were recorded while short, standardized sentences^43,44^ were read aloud from a screen at three different times: Before training (T1), directly after training (T2), and 4 weeks after the end of training (T3).

#### Texts used in the examinations at T1, T2, T3 (Fig. 2)

**Figure 2:**
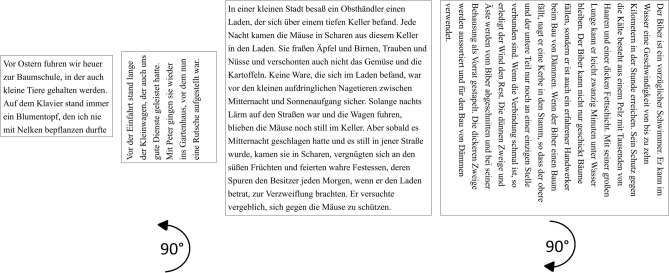
Text material. The left graphs show examples of the short sentences (Radner) displayed for reading on the screen during EM recording. The right graphs show examples of the IReST paragraphs for reading printed text, which were presented as single lines. Text material was presented horizontally or vertically, rotated in counterclockwise (short text left) or clockwise direction (paragraph right) – depending on the patient’s preference. All 21 patients read the texts in horizontal orientation at all times. Only the patients with vertical training read the texts also in vertical direction.

Short, standardized sentences (Radner^43^: 14 words, 3 lines each; set in 12pt Times New Roman) were presented on the screen during eye tracking. Two sentences were shown together in order to provide 6 lines of text with 5 line-return sweeps. For reading print, one standardized text paragraph (IReST, set in 1.2 M Times New Roman)^45,46^ (130 words, 15 lines) was presented line by line, either in horizonal or in vertical orientation. We use the terms “orientation” for the text (0°, 270° or 90°) and the term “direction” for the reading process during training and during examinations in the clinic. Both types of text were presented in horizontal orientation to all patients, in vertical orientation only to the patients in the vertical training group V. A new, but linguistically equivalent, text was used for each examination to avoid repetition, which prevented memorizing the content.

### Outcome variables

#### Reading speed (RS)

Reading speed during reading sentences from a screen during eye tracking (RS-screen):

Two short Radner sentences on a screen were read aloud in the clinic during eye tracking, either in horizontal (RS-screen-H) or in vertical text orientation (RS-screen-V) at the three time points. It was calculated as reading time (measured by the eye tracking software) divided by the number of read words (2*14 words) multiplied by 60.

In contrast, in our previous paper^36^ we assessed reading speed during the training at the computer (cRS) at home, which was measured by the training software. Furthermore, we measured reading speed during reading printed paragraphs aloud (RS-print) at the three time points in the clinic**.** RS-print was the main outcome variable and showed significant improvement after training, which was specific for each training group and HFD-side. Furthermore, there was a high positive correlation between RS-print and cRS. This showed that reading performance after training on a screen can be applied to everyday reading of printed text, because it transferred to everyday life.

Therefore, we use RS-print, which was already determined in our previous paper in the same cohort of patients, in the current study again to test its correlation with RS-screen, which could indicate whether training effects of the examined EM variables tested in the current paradigm transfer to everyday life. To derive RS-print in words per minute (wpm), the reading time for each paragraph was measured by stopwatch and was then divided by the number of correctly read words and multiplied by 60.

#### EM variables

We analysed the number of forward saccades per word, the number of backward saccades per word and the number of additional steps during the return sweep to reach the beginning of the next line and fixation duration.

#### Definition of eye movement variables

*Forward and backward saccades:* A saccade was defined as a fast eye movement of minimal amplitude of 0.5 deg (the width of the letter “n”) preceded or followed by a fixation of at least 100 ms.

*Additional steps during the return sweep:* Since patients with left HFD have difficulties finding the beginning of the next line, we examined their behavior during the return sweep to the beginning of the next line: We counted the additional steps during the return sweep that were performed until the patient started to read the text on the new line. These additional steps are hypometric search saccades, in contrast to reading saccades (as defined above). The return sweep itself was not included, because it occurs at the end of each line.

*Fixation duration* was used as a measure of processing time. Individual mean fixation duration per word was calculated as

$$Fixation \;duration \left[ s \right] = \frac{Reading\; duration\; of\; the\; text\; \left[ s \right]}{{Number \;of \;fixations}}$$

A fixation was defined as a period without eye movements of =  > 0.5° that was followed or preceded by a saccade and was at least 100 ms long, which neglects the saccade durations. The number of fixations was calculated as the sum of forward and backward saccades.

The eye movement recordings during reading vertical lines of text allowed analyzing RS-screen-V (see above). However, they were not of sufficient quality for detailed analysis of the EM variables, because our eye tracker is less accurate in the vertical direction.

Therefore, we calculated the correlations between the eye movement variables that were analyzed during horizontal reading with the reading speed for printed text in horizontal text orientation (RS-print-H).

### Procedures (see also Table 2 above)

#### Reading speed for sentences read off the screen (RS-screen) during eye tracking

Two short sentences (Radner) were presented on the screen horizontally and vertically. The six lines were displayed together for assessment of the EM during 5 return sweeps per trial.

Different sentences were used for each visit and each task.

In group V, the individually preferred reading orientation of the vertical line was determined during the baseline examination. The two line orientations were from bottom to top with an angle of rotation of 90 deg in the counterclockwise direction (for reading upward), or from top to bottom with an angle of rotation of 90° in the clockwise direction (for reading downward; see Fig. 2, above). Nine of the 11 patients chose the direction from top to bottom.

The patients read aloud, which allows determining the reading time, the mistakes made, and assessing fluency and comprehension. The disadvantage is the higher noise of the EM recordings due to mechanical artifacts.

#### Reading speed for printed paragraphs (RS-print)

During reading printed paragraphs on a chart (IReST), upcoming text lines beyond the actually read line were covered by a sheet of paper by the examiner – corresponding to the text presentation during the training. Furthermore, covering the upcoming lines is a common habit of patients in everyday life, which we recommend in clinical practice, because it makes orientation on the page easier. Patients who had trained reading single horizontal lines of text, read the test paragraphs only in horizontal orientation at the three visits. Patients who had trained reading single lines in vertical orientation, read the paragraphs in vertical and horizontal orientation at the three visits. For each visit and each task, different IReST paragraphs were used, all of which belonged to the same performance category^46^.

#### Training

Single lines of text were presented in either horizontal (group H) or vertical (group V) orientation on a computer screen. The patients could choose texts from different categories which were provided by the software, or they could download any text from the Internet. The training software transformed the text into single lines in the required orientation. The text was then displayed line by line, centered on the screen, either horizontally or vertically from top to bottom, or vice versa. The patients went to the next line pressing a button (right arrow key or space bar), and they could also move backwards line-by-line by pressing the left arrow key. The patients trained at home for 30 min, twice a day, for 5 days a week, for 4 weeks. The training intensity was calculated and documented by the software.

#### Fixation and reading assessed by scanning laser ophthalmoscope (SLO)

We used an SLO (Rodenstock 101, Ottobrunn, Germany) to visualize the exact fixation locus on the stimulus. The SLO has a spatial resolution of 0.1° and a temporal resolution of 50 half- frames per second. The SLO shows the image of the retina and the stimulus simultaneously, which shows the position of the fovea relative to the stimulus without calibration. We used this advantage of the SLO-method to perform a descriptive analysis by visualizing the retinal fixation locus during different tasks and to detect pre-existing or potentially new adaptations, such as eccentric fixation and predictive saccades, which are immediately visible when the patient’s fovea lands outside the line of text. Note that an infrared eye tracker cannot show this process as directly as an SLO. The reason is that the SLO shows absolute time of both, the fixation locus on the text and the beginning of vocalization, which can be monitored by the soundtrack.

Since the SLO-examination can be performed only monocularly, we always examined the dominant eye, which was determined by a pin-hole test^47^.

The fixation locus – central or eccentric—was assessed:

- During fixation of a cross (20 s)
- During reading a short sentence of 3 lines, 14 words, using the standardized Radner sentences (Times New Roman, size 1.6 M and 2.5 M). In the SLO, we used only one Radner sentence per screen in order to create a well centered text field in the SLO image to prevent shadowing artifacts that could be caused by temporary misalignment of the eye relative to the instrument.

We observed the landing positions of the fovea within and outside the text to detect potential eccentric fixations and/or dysmetric saccades.

##### Dysmetric saccades

Note that the problem of the limited perceptual span during reading is only present in horizontal reading due to the vertical splitting of the visual field in HFD. Hypometric saccades to find the next line in left HFD or finding the line end in right HFD are time-consuming^39,41^. The HFD patient could overcome the lack of text preview by developing hypermetric (overshooting) and, in the best of cases, predictive saccades during horizontal reading. Therefore, we aimed to examine whether such adaptations would actually happen during horizontal reading in patients with HFD.

### Statistics

Data were analyzed using IBM SPSS statistics (IBM Corp. Released 2017. IBM SPSS Statistics for Windows, Version 25.0. Armonk, NY: IBM Corp.). For the mostly not normally distributed EM variables we applied non-parametric tests: Friedmann-test, post-hoc Wilcoxon signed rank test without Bonferroni correction, because it is a closed test procedure^48^. We applied parametric tests for the correlations (Pearson correlation coefficient r), if the variables were normally distributed. In the other cases, we calculated the Spearman correlation coefficient rho.

The required level of significance α for all tests was set at 0.05 (two-sided). Unless otherwise stated, the Shapiro–Wilk normality test and graphical Q–Q plots were used to determine the shape of the distributions. The data were analyzed by training group (V vs. H) and by the side of the HFD (left vs. right). The statistical values are shown in Tables 3 and 4.

### Ethical approval and consent to participate

We confirm that all experiments were performed in accordance with relevant guidelines and regulations. The project was approved by the ethics committee of the medical faculty of the University of Tübingen, Germany (Reference number: 066/2016B02) and written informed consent was obtained directly from the patients. This study was conducted in agreement with the tenets of the declaration of Helsinki. The study was registered in the German Clinical Trials register (DRKS-ID: DRKS00018843, March 13th, 2020).

## Results

To enhance readability, the text only conveys the rounded medians and the significant p-values, while all other statistics are shown in Tables 3 and 4.

### Reading speed while reading off the screen during eye tracking (RS-screen)

#### Horizontal reading

RS-screen-H improved in both groups and sides, but the improvement did not reach statistical significance:

For the entire cohort, RS-screen-H improved from T1 to T2 (medians 101–111 wpm.

**Figure 3:**
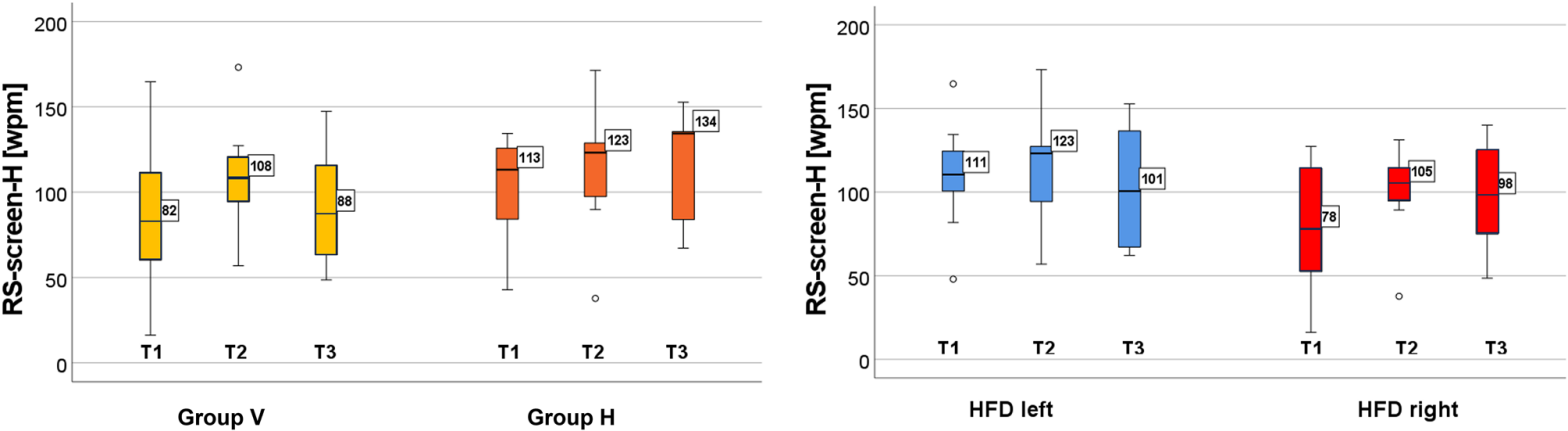
Reading speed during reading in horizontal direction on the screen during eye tracking (RS-screen). Increase of RS-screen from T1 to T2 in both groups (left graph) and in left and right HFD (right graph), however not reaching significance.

If the cohort was separated by training group, RS-screen-H increased from T1–T2 in both training groups (group H: 113–123 wpm; group V: 82–108 wpm; Fig. 3 left). In group H, RS remained stable or even increased further at T3. If they were separated by the side of the HDF, both sub-groups of patients showed an improvement (left: 111–123 wpm, right: 78–105 wpm; Fig. 3 right). (Fig. 3 and Table 3).

**Table 3:** Statistical data: Reading Speed values and correlations.

RS	T1	T2	T3	Tests
	Median (IQR)			
RS-screen horizontal [wpm]				
All patients (n = 19;17;17)	100.6 (67.98–121.74)	110.53 (92.11–126.79)	100.6 (71.10–135.49)	ft(T1–T2–T3): χ^2^(2) = 4.980, *p* = .083
Group H (n = 8;8;7)	113.19 (79.10–126.57)	123.19 (93.63–130.02)	134.4 (78.50–136.59)	ft(T1–T2–T3): χ^2^(2) = 2.800, *p* = .247
Group V (n = 11;9;10)	81.95 (52.50–114.29)	108.39 (91.87–124.07)	87.8 (63.06–121.90)	ft(T1–T2–T3): χ^2^(2) = 4.710, *p* = .095
HFD left (n = 9;10;9)	110.53 (91.21–129.42)	123.19 (93.25–138.31)	100.6 (65.27–141.98)	ft(T1–T2–T3): χ^2^(2) = 2.516, *p* = .284
HFD right (n = 10;7;8)	77.98 (50.09–116.15)	105 (89.36–120.86)	97.85 (75.00–129.77)	ft(T1–T2–T3): χ^2^(2) = 2.800, *p* = .247
RS-print-H horizontal [wpm]				
All patients, n = 21	112 (66.75–124.00)	125 (87.25–143.40)	123 (73.80–138.50)	ft(T1-T2-T3): χ^2^(2) = 8.667, ***p* = .013**
				w(T1–T2): Z = − 0.762, ***p* = .014**; w(T1-T3): Z = − 0.810, ***p* = .009**; w(T2–T3): Z = − 0.48, *p* = .877
Group V, n = 11	99 (63.00–128.00)	99.28 (77.00–143.00)	115 (73.60–131.40)	ft(T1–T2–T3): χ^2^(2) = 1.273, *p* = .529
Group H, n = 10	112.5 (67.38–123.00)	127.4 (104.00–144.78)	124.5 (71.50–144.56)	ft(T1–T2–T3): χ^2^(2) = 9.800, ***p* = .007**
				w(T1–T2): Z = − 1.10, ***p* = .014**; w(T1–T3): Z = − 1.30, ***p* = .004**; w(T2–T3): Z = − 0.200, *p* = .655
HFD left, n = 11	113 (80.00–120.00)	129.8 (99.28–144.00)	126 (82.50–141.00)	ft(T1–T2–T3): χ^2^(2) = 7.818, ***p* = .020**
				w(T1–T2): Z = − 1.182, ***p* = .006**; w(T1–T3): Z = − 0.727, *p* = .088; w(T2–T3): Z = 0.455, *p* = .286
HFD right, n = 10	104.5 (47.78–129.00)	109.75 (57.23–143.20)	119 (59.00–130.35)	ft(T1–T2–T3): χ^2^(2) = 4.200, *p* = .122
RS-screen vertical [wpm])				
All patients of group V (n = 11)	74.67 (47.95–96.49)	100 (45.41–111.26)	92.31 (47.59–100.00)	ft(T1–T2–T3): χ^2^(2) = 7.800, ***p* = .020** w(T1–T2): Z = − 1.200, ***p* = .007**; w(T1–T3): Z = − .900, ***p* = .044**; w(T2–T3): Z = .300, *p* = .502
HFD left (n = 4;5,5)	73.46 (55.67–96.10)	71.19 (50.57–115.63)	73.68 (42.93–101.12)	ft(T1–T2–T3): χ^2^(2) = 1.500, *p* = .472
HFD right (n = 6;6;6)	75.67 (28.09–96.80)	102.83 (38.87–107.93)	93.88 (50.07–100.61)	ft(T1–T2–T3): χ^2^(2) = 7.000, ***p* = .030** w(T1–T2): Z = − 1.500, ***p* = .009**; w(T1–T3): Z = − 1.000, *p* = .083; w(T2–T3): Z = − .500, *p* = .386
RS-print-V vertical [wpm]				Friedmann-Test, Post-hoc Wilcoxon signed rank test
All patients of group V, n = 11	90 (44.56–112.00)	104 (56.50–116.40)	104 (58.20–111.00)	ft(T1–T2–T3): χ^2^(2) = 14.000, ***p* < .001** w(T1–T2): Z = − 1.318, ***p* = .002**; w(T1–T3): Z = − 1.409, ***p* < .001**; w(T2-T3): Z = − 0.091, *p* = .831
HFD left, n = 5	70 (43.03–116.00)	77 (57.51–130.70)	73 (58.80–121.20)	ft(T1–T2–T3): χ^2^(2) = 6.400, ***p* = .041** w(T1–T2): Z = − 1.600, ***p* = .011**; w(T1–T3): Z = − 0.800, *p* = .206; w(T2-T3): Z = 0.800, *p* = .618
HFD right, n = 6	93.85 (46.66–102.04)	104.5 (47.44–110.25)	104.7 (49.21–116.25)	ft(T1–T2–T3): χ^2^(2) = 11.565, ***p* = .003** w(T1–T2): Z = − 1.083, **p < .001**; w(T1–T3): Z = − 1.917, *p* = .149; w(T2-T3): Z = − 0.833, *p* = .180
Correlations				
RS-print – RS-screen (both vertical)	Pearson's r	Pearson's r	Pearson's r	
All patients	**.933** (n = 10, ***p* < .001)**	**.946** (n = 11, ***p* < .001)**	**.911** (n = 11,** p < .001)**	
HFD left	**.908** (n = 4, *p* = .092)	**.921** (n = 5, ***p* = .026**)	**.883** (n = 5, **p = .047**)	
HFD right	**.981** (n = 6, ***p* < .001**)	**.976** (n = 6, ***p* = .026**)	**.935** (n = 6, **p = .006**)	
RS-print-H – RS-screen-H	Pearson's r	Pearson's r	Pearson's r	
All patients	**.909** (n = 21, ***p* < .001)**	**.845** (n = 17, ***p* < .001)**	**.772** (n = 17, ***p* < .001)**	
HFD left	**.916** (n = 9, *p* < .001)	**.881** (n = 10, *p* < .001)	**.704** (n = 9, *p* = .034)	
HFD right	**.945** (n = 10, *p* < .001)	**.927** (n = 7, *p* = .003)	**.868** (n = 8, *p* = .005)	

All procedures and measurements of analyzed data.

*V* vertical training group, *H* horizontal training group; *wpm* words per minute; *HFD* hemianopic field defect; *IQR* interquartile range; *ft* Friedman test; *w* post hoc Wilcoxon signed-rank test; *Z* test-statistics (Wilcoxion signed rank test), no Bonferroni correction (closed testing procedure), *n* number of patients, *p* significance value.

The values of RS-print are from our previous paper^36^.

Reading speed of printed paragraphs (IReST), in horizontal (RS-print-H) and vertical (RS-print-V) direction and reading speed of short sentences on the screen during eye tracking (Radner) in horizontal (RS-screen-H) and vertical (RS-screen-V) direction. Correlations between RS-print and RS-screen.

#### Vertical reading

**Figure 4:**
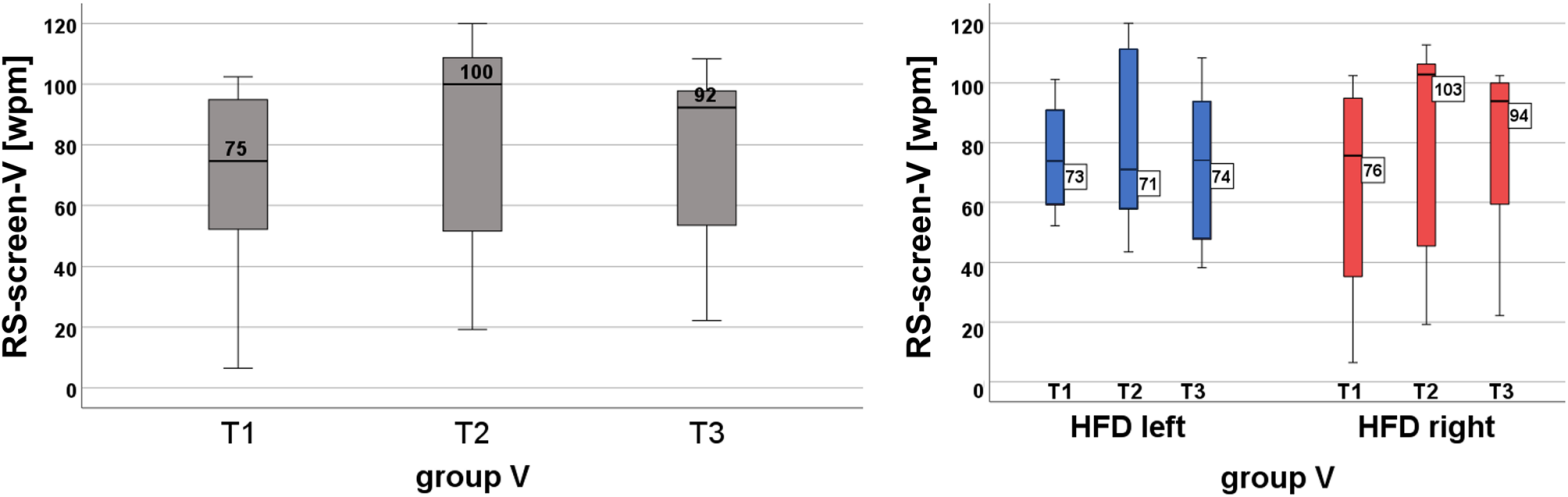
Reading speed during reading in vertical direction on the screen during eye tracking (RS-screen). Significant improvement in the entire group V (left graph) from T1 to T2 and from T1 to T3. Separated by the side of the HFD (right graph): no change in left HFD, significant improvement in right HFD.

The entire group V improved RS-screen-V significantly from T1 to T2 (medians 75–100 wpm, *p* = 0.007) and from T1 to T3 (medians 75–92, *p* = 0.044). The effect remained stable at T3 (see Fig. 4).

Separated according to the side of the HDF, there was no change in those with a left HFD (medians 73–71-74 wpm), but in those with a right HFD, RS-screen-V improved significantly from T1 to T2 (medians 76–103 wpm, *p* = 0.009). The effect remained stable at T3. This shows the specific effect of vertical training on vertical reading speed.

### Correlation between RS-screen and RS-print (Fig. 5 and Table 3)

**Figure 5:**
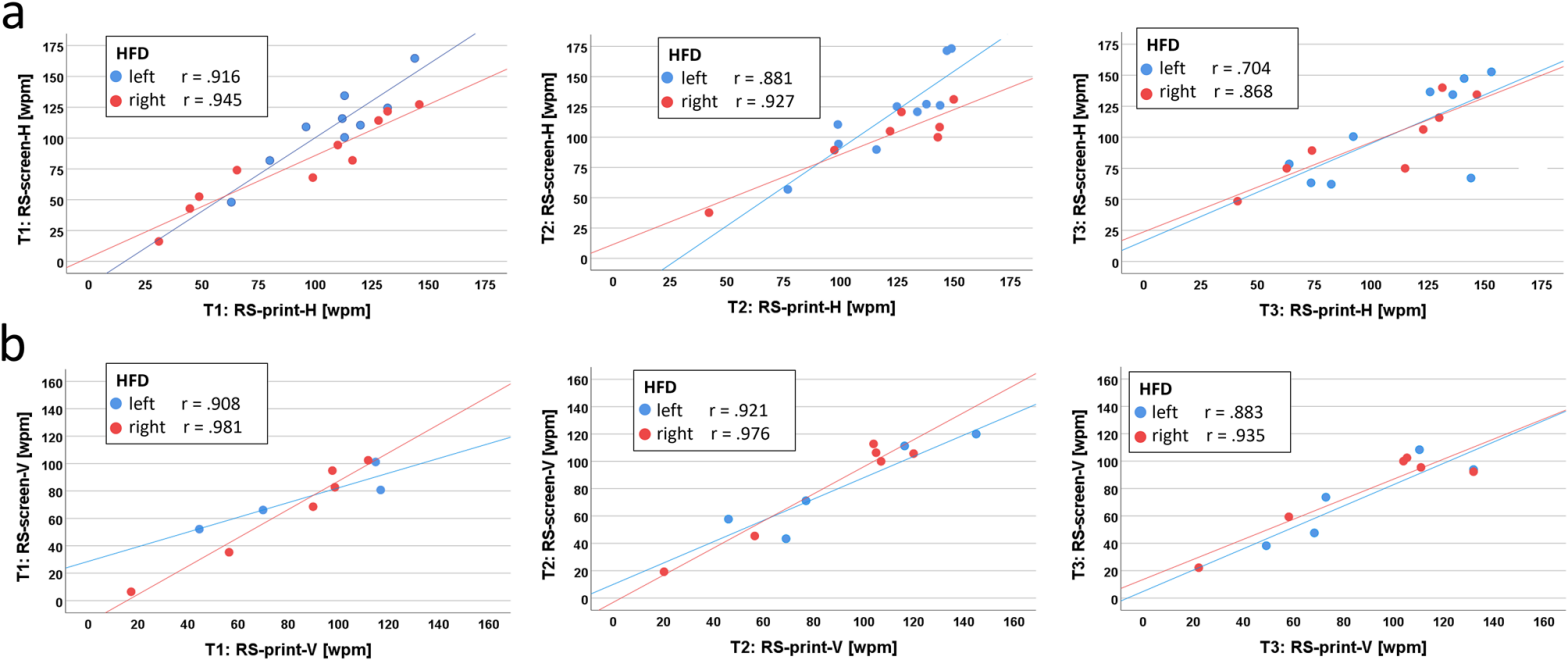
Correlation between RS-print and RS-screen. (a) in horizontal text orientation, (b) in vertical text orientation. High positive correlation between RS-print and RS-screen in both orientations and at all three time points. Regression lines added.

#### Horizontal reading

For the entire cohort, there was a high positive correlation between RS-print-H and RS-screen-H (T1: r = 0.909, *p* < 0.001; T2: r = 0.845, *p* < 0.001; T3: r = 0.772, *p* < 0.001).

Separated according to the side of the HFD, there was a high positive correlation between RS-print-H and RS-screen-H for left and right HFD patients at all 3 time points (left HFD: T1: r = 0.916, *p* < 0.001; T2: r = 0.881, *p* < 0.001; T3: r = 0.704, *p* = 0.034 ; right HFD: T1: r = 0.945, *p* < 0.001; T2: r = 0.927, *p* = 0.003, T3: r = 0.868, *p* = 0.005) (Fig. 5a).

#### Vertical reading

For the entire group V, there was a high positive correlation between RS-print-V and RS-screen-V at all 3 time points (T1: r = 0.933, *p* < 0.001; T2: r = 0.946, *p* < 0.001; T3: r = 0.911, *p* < 0.001) – see Fig. 5b. The high correlation coefficients were statistically significant for left HFD at T2 and T3 (T1: r = 0.981, *p* = 0.92; T2: r = 0.921, *p* = 0.026; T3: r = 0.883, *p* = 0.047) and for right HFD at all 3 time points (T1: r = 0.981, *p* < 0.001; T2: r = 0.976, *p* = 0.026; T3: r = 0.935, *p* = 0.006).

These high positive correlations between reading speeds of printed text and on the screen in horizontal and vertical reading at all 3 time points showed the excellent comparability of the results. This enabled us to focus on the correlations between RS-print and the specific EM variables in the following analysis. We chose RS-print, because this is the measure of the transfer to daily life. As the EM variables could only be analyzed for horizontal reading, the horizontal reading speed of printed text (RS-print-H) was used for the calculations of correlations.

The typical reading problem is different in left and right HFD. For patients with a left HFD, the problem is to find the next line. Those with a right HFD have to make many small saccades in reading direction. Therefore, the training effect on the EM variables is also expected to be different, and we will focus on the results dependent on the side of the HFD (below).

### Specific EM variables

#### Forward saccades per word (Fig. 6 and Table 4)

**Figure 6:**
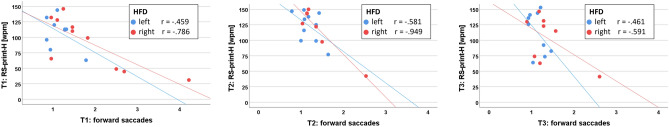
Correlation between RS-print and number of forward saccades per word. High negative correlation between forward saccades and RS-print for right HFD at T1 and T2. Right HFD-patients start with a higher number compared with the left HFD-patients. No correlation for left HFD. This shows that in right HFD the improvement of RS is caused by the decrease of the number of forward saccades per word. Regression lines were added.

The mean word length of the Radner sentences was 5 letters (range: 2–11). In left HFD, there was no change between the time points (1.11–1.13–1.14 saccades/word), but in right HFD, the number of forward saccades decreased from 1.46 (T1) to 1.18 (T2) – without reaching statistical significance (Table 4).

**Table 4:** Statistical data. EM-Variables.

EM-Variables	T1	T2	T3	Tests
	Median (IQR)			
Forward saccades				
HFD left (n = 9;10;9)	45,231	41,275	41,640	ft(T1–T2–T3): χ^2^(2) = 3.161, *p* = .206
	(0.90–1.30)	(1.05–1.38)	(0.95–1.30)	
HFD right (n = 10;7;8)	16,803	43,101	45,292	ft(T1–T2–T3): χ^2^(2) = 0.737, *p* = .692
	(1.07–2.55)	(1.14–1.50)	(1.10–1.50)	
Backward saccades				
HFD left (n = 9;10;9)	0.61	0.60	0.71	ft(T1–T2–T3): χ^2^(2) = 2.467, *p* = .291
	(0.46–0.87)	(0.49–0.74)	(0.45–1.16)	
HFD right (n = 10;7;8)	0.77	0.50	0.63	ft(T1–T2–T3): χ^2^(2) = 4.562, *p* = .104
	(0.43–1.08)	(0.39–0.75)	(0.50–0.87)	
Additional steps during return sweep				
HFD left (n = 9;10;9)	45,047	1.00	42,736	ft(T1–T2–T3): χ^2^(2) = 4.067, *p* = .131
	(0.67–2.00)	(0.33–1.42 )	(0.67–1.83)	
HFD right (n = 10;7;8)	0.63	0.50	0.33	ft(T1–T2–T3): χ^2^(2) = 0.105, *p* = .949
	(0.42–0.88)	(1.67–0.67)	(1.67–1.08)	
Fixation duration				
HFD left (n = 9;10;9)	0.30	0.27	0.30	ft(T1–T2–T3): χ^2^(2) = 6.750, ***p* = .034**
	(0.27–0.35)	(0.25–0.32)	(0.29–0.37)	w(T1–T2): Z = 1.125, ***p* = .024**;
				w(T1–T3): Z = 0.000, *p* = 1.000**;**
				w(T2–T3): Z = − 1.125, ***p* = .024**
HFD right (n = 10;7;8)	0.34	0.34	0.33	ft(T1–T2–T3): χ^2^(2) = 0.000, *p* = 1.000
	(0.31–0.37)	(0.30–0.36)	(0.29–0.37)	
Correlations				
RS-print-H – forward saccades	Pearson's r	Pearson's r	Pearson's r	
HFD left	− .459 (n = 9, *p* = .214)	− .581 (n = 10, *p* = .101)	− .461 (n = 9, *p* = .211)	
HFD right	− .786 (n = 10, *p* = .007)	− .949 (n = 7, *p* = .001)	− .591 (n = 8, *p* = .123)	
RS-print-H – backward saccades	Spearman-Rho	Spearman-Rho	Pearson's r	
HFD left	Rho = − .678 (n = 9, *p* = .045)	Rho = − .806 (n = 10, *p* = .005)	− .555 (n = 9, *p* = .121)	
HFD right	Rho = − .912 (n = 10, *p* < .001)	Rho = − .865 (n = 7, *p* = .012)	− .770 (n = 8, *p* = .025)	
RS-print-H – additional steps				
During return sweep	Pearson's r/Spearman-Rho	Pearson's r/Spearman-Rho	Pearson's r/Spearman-Rho	
HFD left	r = − .833 (n = 9, *p* = .005)	Rho = − .561 (n = 10, *p* = .092)	r = − .729 (n = 9, *p* = .026)	
HFD right	Rho = − .738 (n = 10, *p* = .015)	r = − .727 (n = 7, *p* = .064)	Rho = − .405 (n = 8, *p* = .319)	
RS-print-H – fixation duration	Pearson's r	Pearson's r	Pearson's r	
HFD left	− .551 (n = 9, *p* = .124)	− .635 (n = 10, *p* = .048)	− .596 (n = 9, *p* = .090)	
HFD right	− .679 (n = 10, *p* = .031)	− .607 (n = 7, *p* = .149)	− .570 (n = 8, *p* = .140)	

*V* vertical training group, *H* horizontal training group; *HFD* hemianopic field defect; *IQR* interquartile range; *ft* Friedman test; *w* post hoc Wilcoxon signed-rank test; *Z* test-statistics (Wilcoxon signed rank test), *n* number of cases, *p* significance value. *Correlations* correlations between reading speed of printed text (RS-print) horizontally with the EM variables; *n* number of patients. *Spearman Rho* Spearman’s rank correlation coefficient (Spearman's ρ), *Pearson r* Pearson product-moment correlation coefficient.

In cases where the data were non-normally distributed, Spearman’s Rho was calculated, otherwise Pearson’s r.

However, the correlation between forward saccades and RS-print-H showed high negative correlations for right HFD at T1 (r =  − 0.786, *p* = 0.007) and T2 (r =  − 0.949, *p* = 0.001). Right HFD-patients start with a higher number of forward saccades compared with the left HFD-patients (Fig. 6 and Table 4). There was only a moderate negative correlation for left HFD (T1: r =  − 0.459; T2: r =  − 0.581, T3: r =  − 0.461), without statistical significance.

This shows that in patients with a right HFD, the improvement of RS is caused by the decrease of the number of forward saccades per word.

#### Backward saccades per word (Fig. 7)

**Figure 7:**
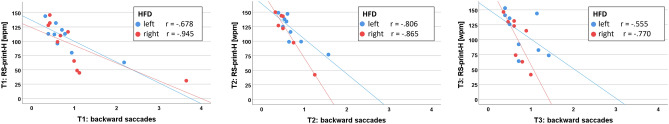
Correlation between RS-print and number of backward saccades per word. High negative correlation of backward saccades with RS-print at T1 and T2, stronger for right HFD. The decrease of the number of backward saccades influences the RS-improvement in all patients, but more in right HFD-patients, who start with a higher number of backward saccades. Regression lines were added.

There was no change from T1 to T2 in patients with left HFD (0.61–0.6), but a decrease in those with right HFD (from 0.77 to 0.5), without reaching statistical significance (see Table 4). However, the negative correlation of backward saccades with RS-print-H was high at T1 and T2, and stronger for patients with right HFD (left HFD: T1: rho =  − 0.678, *p* = 0.045; T2: rho = -0.806, *p* = 0.005; T3: r = 0.555, *p* = 0.121; right HFD: T1: rho =  − 0.912, *p* < 0.001; T2: rho =  − 0.865, *p* = 0.012, T3: r = 0.770, *p* = 0.025) (Fig. 7 and Table 4).

The decrease of the number of backward saccades thus influenced the improvement of RS in all patients, but more in those with right HFD, who start with a higher number of backward saccades (Fig. 7 and Table 4).

#### Additional steps during the return sweep (Fig. 8)

**Figure 8:**
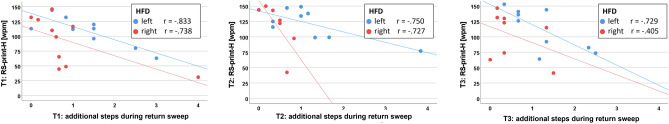
Correlation between RS-print and number of additional saccadic steps during the return sweep. High negative correlation for left HFD, no correlation for right HFD. This shows that in left HFD the improvement of RS is caused by the decrease of the number of additional backward saccades during the return sweep. Regression lines were added.

Additional steps during the return sweep decreased from T1 to T2, which was more pronounced in cases with left (1.5–1.0) than in those with right HFD (0.63–0.50) but did not reach statistical significance (Table 4). However, the correlations between the number of additional steps during the return sweep and RS-print-H showed a high negative correlation for patients with left HFD at T1 and T3 (T1: rho =  − 0.833, *p* = 0.005; T2: r =  − 0.561, *p* = 0.092; T3: r =  − 0.729, *p* = 0.026), and a negative correlation in right HFD only at T1 (T1: rho =  − 0.738, *p* = 0.015; T2: r =  − 0.727, *p* = 0.064; T3: rho =  − 0.405, *p* = 0.319) (Fig. 8 and Table 4).

In cases with left HFD, the improvement of RS is caused by the reduced number of additional steps during the return sweep.

#### Fixation duration

The fixation duration decreased statistically significantly during training, but only slightly, in patients with left HFD (from 0.30 ms at T1 to 0.27 ms at T2. *p* = .024), and it increases again from T2 to T3 (0.30 ms). There was a moderate negative correlation between fixation duration and RS-print -H in patients with left HFD at T2 (r =  − 0.635, *p* = 0.048). In right HFD, there was no change of fixation duration (T1: 0.34, T2: 0.34, T3: 0.33 ms) and only a moderate correlation with RS-print-H at T1 (r =  − 679, *p* = 0.031), see Table 4. We conclude that fixation duration contributes to the improvement of RS only slightly and only in patients with a left HFD**.**

### Training intensity

The required training time was 2 × 30 min per day, 5 days per week, for 4 weeks (1200 min = 20h). The cumulative results showed a median training time for all patients of 1236.45 min (IQR 872.2–1417.4, range 715.48 – 1963.1 min) (103%). The median training time per day was 62 min, this means that there was on average good compliance.

### Fixation and reading assessed by SLO

#### Fixation locus while fixating a cross for 20 s

Our focus was to examine whether the retinal fixation locus was the fovea (central, as in healthy subjects and in patients with HFD with macular sparing) or outside the fovea (paracentral, eccentric), which is of significance for reading (see above), and whether it changed during the training.

**Figure 9:**
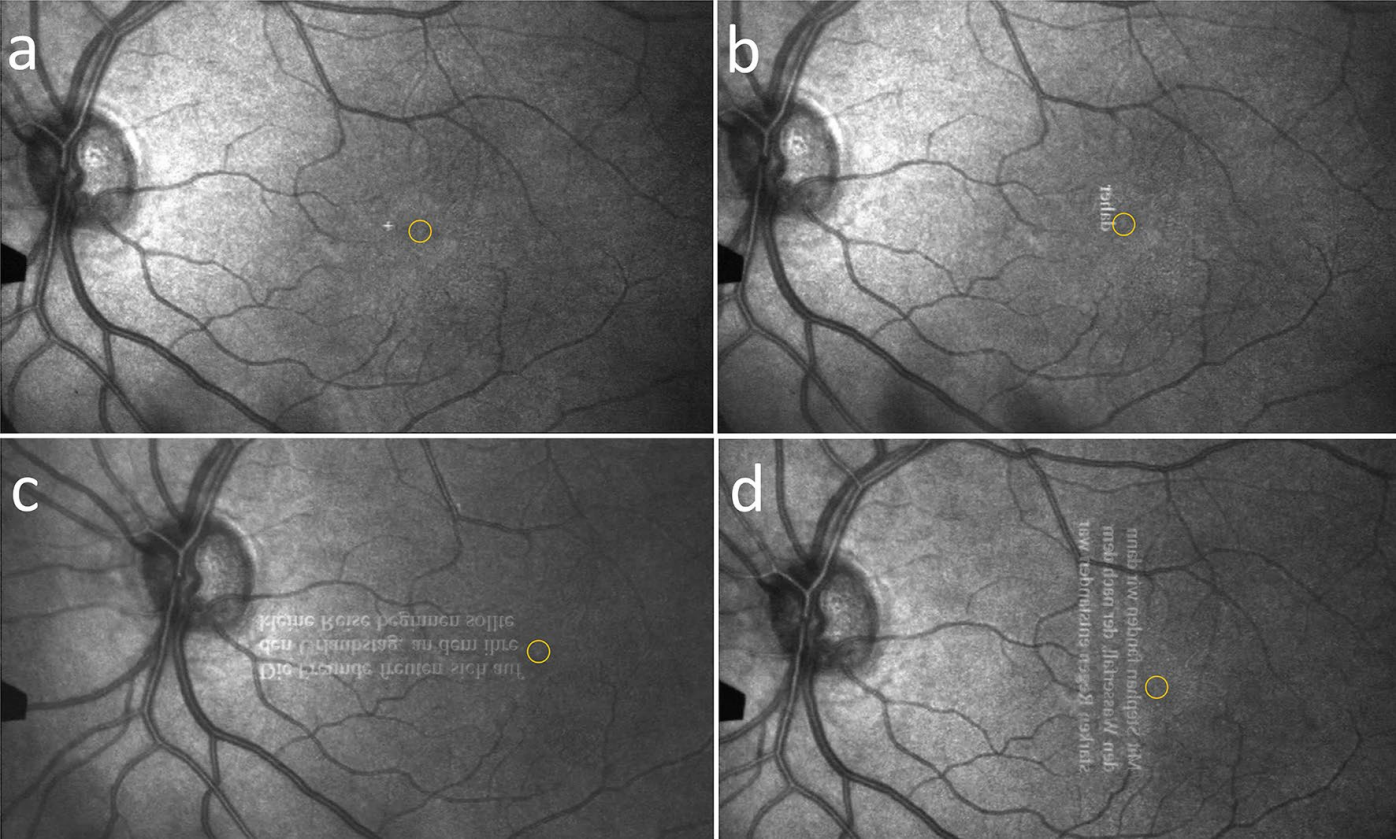
SLO images of a right HFD patient’s retina with eccentric fixation. (**a**) eccentric fixation of a cross, (**b**) reading a single word in vertical direction. By using a slightly eccentric retinal locus she creates a small perceptual area along the vertical midline, thus placing the line optimally in the seeing visual hemifield. (**c**) reading a short sentence in horizontal orientation. The text appears upside down for the examiner, but upright for the patient. The image shows an example of the foveola (yellow circle) landing outside the end of the line. This is a favorable spontaneous adaptive behavior that indicates that the benefit by shifting the text into the seeing hemifield existed before the training. It can be caused by a hypermetric saccade and eccentric fixation during reading. (**d**) reading a short sentence in vertical direction. The lines are not limited by the scotoma. The patient reads the first line (right) with her eccentric fixation locus and shifts the line into her seeing hemifield.

At T1, ten patients fixated centrally with occasional short saccades of 1–2° towards the blind hemifield; 10 patients changed between central and eccentric fixation (by 1°), i.e. they had not established an eccentric preferred retinal locus while one patient had adopted eccentric fixation (by 1–1.5°), shown in Fig. 9a.

At T2, only 4/21 patients changed their behavior: Two patients changed their fixation locus between central and eccentric less often, i.e. they showed more stable fixation than at T1. They improved their RS markedly (one patient by 27 wpm, another one by 44.8 wpm). One patient changed from central to eccentric fixation, one patient vice versa—both without improvement of RS. Therefore, the training did not have a general effect on fixation of a cross.

#### Fixation locus during text reading

Twenty patients fixated the texts centrally in either text orientation. One patient with right HFD in the vertical training group had already adopted eccentric fixation before training and gained a 1–1.5° perceptive area along the vertical midline, which was especially beneficial in vertical reading (Fig. 9b, d). She improved RS by the vertical reading training markedly, i.e. by 17 wpm from T1 to T2 and by 21 wpm from T1 to T3 (90/107/111 wpm). During horizontal reading, RS did not change (128/127/130 wpm), but was higher than in vertical reading. This patient’s disease duration was 26 years, so that she was already adapted to using an eccentric fixation locus during horizontal reading. This example shows that vertical reading in combination with using an eccentric retinal locus provides improved sensory conditions for reading.

#### Fovea landing (dysmetric saccades) during text reading

The aim of this analysis was to describe reading strategies by observing where the patients’ fovea landed on the horizontal text in order to identify their reading problem by hypometric saccades at the line beginning (in left HFD) or line end (in right HFD), as well as potential adaptive behaviors by hypermetric and predictive saccades.

At T1, we analyzed the pre-existing pattern of dysmetric saccades at the line beginning for left HFD and at the line end for right HFD.

During reading 3 lines of text horizontally, the patients showed the typical eye movement patterns dependent on the side of the HFD.

##### Patients with left HFD (n = 11)

At T1, eight out of 11 patients did not show adaptive behavior: The patients often missed the beginning of a word within the line and the beginning of the next line. During the return sweep we observed different behaviors: In some cases, the patient`s fovea landed near to the beginning of the next line, which was followed by several backward steps until the beginning was found. In other cases, the patient´s fovea landed within the 2nd or 3rd word of the next line, the patient made 1 or 2 forward and backward saccades, then realized that this was not the correct continuation of the context, and then made another return sweep to the beginning of the line. These hypometric saccades are ineffective and time-consuming. We saw in 3 patients the fovea landing just left of the first letter by a predictive saccade^39,41^, probably supported by eccentric fixation. This adaptation provides a preview benefit for finding the beginning of the next line.

At T2; here, we examined whether the patients changed their behavior of dysmetric saccades. Four out of 6 patients of the horizontal training group and 2 out of 5 in the vertical training group had reduced their dysmetric saccade behavior or enhanced their overshooting or predictive strategy. The improved landing positions were not associated with the increase of RS-print-H, which shows that the improvement of RS-print-H during horizontal reading was primarily due to the improved EM by less additional steps during the return sweep by the horizontal training.

##### Patients with right HFD (n = 10)

*A1 T1:* Four out of 10 patients did not show an adaptive behavior: The patients had to go through the line with many short forward and backward saccades. At T1, four patients landed within the last word of the line. Six patients had adopted an adaptive behavior, when the fovea landed outside the end of the line. An example is shown in Fig. 9c.

At T2: All 4 patients in the horizontal training group improved their dysmetric or predictive behavior. Of the 6 patients in the vertical training group, only one patient improved the predictive saccades at the line end during horizontal reading, five did not show any change of their pre-existing behavior. In all patients with right HFD, there was no association with the increase of RS-print-H in horizontal reading. It shows that the increase of RS-print in right HFD is caused by the vertical reading training. For the patients having trained reading in vertical text orientation, no improvement of RS-print-H in horizontal direction is to be expected.

## Summary of the results

- The training effect was specific for the reading direction: Vertical training improved RS-screen and RS-print only for vertical reading, horizontal training improved RS-screen and RS-print only for horizontal reading. All RS-improvements were statistically significant, except for RS-screen horizontal. The effects of the EM variables after training depended on the side of the HFD: In patients with right HFD, the improvement of RS-print was caused by the decrease of the number of forward saccades per word and to a lesser degree by a lower number of backward saccades. In cases with left HFD, the improvement of RS-print was primarily caused by the decrease of the number of additional saccadic steps during the return sweep, which takes less time. A lower number of backward saccades and a shorter fixation duration further contributed to the improvement.
- However, RS during vertical reading did not reach the same level as during horizontal reading.
- Transfer of the training benefit to daily life is indicated by the stable effect at T3 and the high positive correlation between RS-screen and RS-print despite having trained on a screen. The majority of the patients showed an adaptive behavior of various degrees before the training. At T2, 8 of 10 patients of group H and 2 of 10 patients of group V showed a slightly more advanced adaptation.

## Discussion

Previous studies on healthy persons with simulated hemianopia^18^ and studies on patients with hemianopia, but without training^34,35^, assumed a potential benefit by vertical reading training, although reading was slower in vertical text orientation. Whereas rotating the text will not be of advantage to individuals without visual field defect, patients with HFD can benefit from the improved sensory input, because their perceptual span will not be limited by the scotoma.

In the current study, we found statistically significant improvement of reading speed in the trained reading direction. This means that the training had a specific effect. Even though the improvement of RS-screen-H did not reach statistical significance, the increase of RS-screen-H of =  > 10 wpm is considered clinically relevant.

In addition, the side of the HFD played an important role: Patients with right HFD benefited from vertical reading training, in contrast to patients with left HFD, who improved their reading speed during horizontal training. This agrees with the findings by DeJong and coworkers that in left HFD, the text rotation was not helpful, whereas in right HFD the smallest speed reduction occurred with 90° clockwise rotated texts and without previous training^34^. In contrast, patients with right HFD are more disabled, because their scotoma is in reading direction and thus, they need to make many small saccades to get through the line^1–7^. It is conceivable that they can benefit most if the limitation of their perceptual span is abolished by reading along the vertical midline and placing the text line in the seeing hemifield. In contrast, patients with left HFD have a problem finding the beginning of the next line, which is evidenced by making additional saccadic steps during the return sweep^5–7,9^. RS-screen was highly correlated with RS-print in vertical and horizontal text orientation. As RS-print is a measure of the transfer to daily life, we looked for correlations between the EM variables and RS-print.

The EM variables improved dependent on the side of the HFD:

In right HFD, RS improved only in the vertical training group and was primarily caused by the decrease of the number of forward saccades, and less by a decrease of the number of backward saccades. This beneficial effect concerns the primary difficulty of getting through the line in patients with right HFD.

In left HFD, RS improved only in the horizontal training group. This is primarily caused by the decreased number of additional steps during the return sweep that affects the primary difficulty to find the beginning of the next line. The decrease of fixation duration further contributes to the improvement.

We used the SLO to visualize the fovea landing at the line beginning in left HFD and at the line end in right HFD and looked for potential adaptations before and after the training. The fixation locus (central or eccentric) was not influenced by the training. However, eccentric reading along the vertical midline is especially beneficial, because it allows shifting the line to be read into the seeing hemifield.

Hypermetric and predictive saccades occur when the fovea lands outside the beginning of the line in patients with left HFD and outside the line end in patients with right HFD. This is a favorable spontaneous adaption from which the patient benefits and which may have existed already before the training. The majority of our patients showed such adaptions of various degrees before the training. After the training, eight of 10 patients of group H and 2 of 10 of group V showed a slightly more advanced adaptation. This could have been supported by horizontal training. It is conceivable that such adaptations could be supported by specific oculomotor training^8^. It is possible that the vertical training had no effect, because the line beginnings and ends are always visible here. Figure 9 c shows an example for the fovea landing outside the end of the line. This is a favorable spontaneous adaptive behavior that indicates that the benefit by shifting the text into the seeing hemifield existed before the training. In this patient, this can be caused by both: hypermetric saccadic strategy and eccentric fixation.

Meienberg^41^ and our previous study^39^ found that these spontaneous adaptations are not correlated with the disease duration, which indicates insufficient spontaneous long-term adaptation and demonstrates the demand for specific reading training.

RS during vertical reading did not reach the same level as during horizontal reading. This could have several reasons:

1) In alphabetic languages, which are usually read horizontally, the perceptual span during reading was found to extend farther in the reading direction. This was found for reading from left to right^17,49^, such as English and all other European languages, as well as for languages that are read from right to left, such as Hebrew^50^, Arabic^51^ and Urdu^49^. This shows that the script of a language and the learned reading direction influence the size of the perceptual span in order to create a preview benefit to determine the size of the next saccade.

2) Life-long experience with reading in a horizontal direction in alphabetic scripts not only trains the horizontal reading eye movements, but also the recognition of word shape. Vertical reading saccades and rotated word shapes are unfamiliar and challenging. Even more difficult is the presentation of alphabetic words as “marquee”, where the single letters are written one below the other, which loses the word shape completely and slows reading considerably^23,25,52^.

However, in logographic scripts – such as Japanese and Chinese - the words can be written in both directions without changing their shape - as they keep the same orientation. Each Chinese character represents a word or part of a composed word*.*

It is interesting that in readers who are used to read in both directions - as in Chinese and Japanese - the difference between horizontal and vertical reading performance disappears^53^. However, event-related potentials have shown that the recognition of Chinese characters was delayed in occipito–temporal sites if the orientation of the characters was changed^54^. In a recent study, we found that the meaning of a Chinese character is primarily processed visually^55^. Therefore, changing the character orientation interferes with the logographic nature of the character.

Mongolian script, which is alphabetic, is an exception in that it is conventionally written vertically (from top to bottom, with line progression from left to right), but it can also be read horizontally (from left to right). Horizontal text orientation can be achieved by rotating the vertical text by 90° counterclockwise (opposite to what we did here). This way, the orthographic units (letters, words) are always aligned with the reading direction. A study by Su et al.^56^ found the perceptual span to be similar in both reading directions, which again demonstrates the flexibility of the perceptual span.

Furthermore, the influence of the experienced reading direction was examined in experiments using different tasks: In a product choice task, it has been reported that in Japanese readers` reading direction primed activating the corresponding direction of visual information processing^57^. Another study examined the counting direction in a cross-cultural study and showed that the horizontal and vertical counting direction was strongly influenced by the reading direction during recent exposure and longstanding experience^58^. These results show the significance of an individual`s experience for priming a preferred direction in different tasks.

3) A shorter visual span, where letters are seen clearly, i.e. a lower resolution was reported for the vertical meridian compared with the horizontal one^27–29^. Yu et al.^25^ examined vertical reading using Rapid Serial Visual Presentation (RSVP) to eliminate reading eye movements as well as short sentences on flashcards. RSVP showed a higher reading speed than flashcard presentation. They found that the slower reading speed in the vertical conditions was caused by the shorter visual span and not by the vertical reading eye movements.

The visual span is defined by the retinal resolution based on the cone density, whereas the perceptual span can be increased in reading direction by parafoveal information processing, where the letters are no longer seen completely clearly. The perceptual span is a dynamic feature during reading eye movements^16^ and is flexible and can be trained, as described above.

Some patients with long-standing HFD, who had probably developed spontaneous adaptive strategies, mentioned that they would have welcomed training sooner after onset of their disorder.

The following points can be seen as weaknesses of the current study:

1. The sample size was not big enough to form further subgroups.
2. EM variables could be analyzed only for horizontal reading, since the recordings for vertical reading were not of sufficient quality due to technical reasons.
3. Previous studies on vertical reading with healthy subjects did not show a difference in reading speed, if the text was rotated clockwise or counterclockwise^25,52^. Our patients of group V could choose their preferred rotation of the vertical text before the training. All but two chose to read from top to bottom (both had right HFD) during the training. It might have been better, if all patients with right HFD had read from top to bottom and those with left HFD from bottom to top, because the next line would always appear in the seeing hemifield.

## Conclusions

Based on our data and the studies mentioned above, we believe that the slower vertical reading speed in our study is caused by having to make unfamiliar vertical reading saccades and by the unfamiliar rotated word shapes. It is conceivable that longer and earlier training could overcome or reduce these difficulties by increasing the perceptual span in vertical reading direction and by getting used to rotated word shapes. Future studies should investigate, whether the training effect can thus be enhanced.

The data confirmed our hypotheses by showing that reading in either text orientation was improved specifically by the training, and that the effects on the EM pattern depended on the side of the HFD.

Transfer of the training benefit to daily life is indicated by the stable effect at T3 and the fact that the patients could apply their improved reading performance to reading printed paragraphs despite having trained reading single lines of text on a screen. Thus, we could show that training to read text on a screen transferred to reading printed text in a real-life situation.

## Data Availability

The datasets and raw data which were collected and analysed during this study can be shared and are available from the corresponding author upon reasonable request. For privacy and data protection reasons, it is not possible to make this data publicly available.

## Abbreviations

AMD: Age-related macular degeneration
cRS: Reading speed measured during the training at the computer
EM: Eye movements
Group H: Training group to train reading in horizontal text orientation
Group V: Training group to train reading in vertical text orientation
HFD: Hemianopic field defects
IQR: Interquartile range
IReST: The International Reading Speed Texts; standardized text paragraphs
LogMAR: Logarithm of the Minimum Angle of Resolution
MoCA: Montreal cognitive assessment
RS: Reading speed
RS-print: Reading speed measured while reading printed standardized paragraphs
RS-print-H: Reading speed measured while reading printed standardized paragraphs in horizontal text orientation
RS-print-V: Reading speed measured while reading printed standardized paragraphs in vertical text orientation
RS-screen: Reading speed measured while reading short sentences on a screen
RS-screen-H: Reading speed measured while reading short sentences on a screen in horizontal text orientation
RS-screen-V: Reading speed measured while reading short sentences on a screen in vertical text orientation
RSVP: Rapid Serial Visual Presentation
SLO: Scanning Laser Ophthalmoscope
T1: Time before training
T2: Time directly after the training
T3: Time 4 weeks after end of training
wpm: Words per minute
